# Dynamic RMST curves for survival analysis in clinical trials

**DOI:** 10.1186/s12874-020-01098-5

**Published:** 2020-08-27

**Authors:** Jason J. Z. Liao, G. Frank Liu, Wen-Chi Wu

**Affiliations:** Merck & Co., Inc, North Wales, PA 19454 USA

**Keywords:** Immuno-oncology trial, Survival, RMST, Mixture Weibull, Log-rank test, Estimand

## Abstract

**Background:**

The data from immuno-oncology (IO) therapy trials often show delayed effects, cure rate, crossing hazards, or some mixture of these phenomena. Thus, the proportional hazards (PH) assumption is often violated such that the commonly used log-rank test can be very underpowered. In these trials, the conventional hazard ratio for describing the treatment effect may not be a good estimand due to the lack of an easily understandable interpretation. To overcome this challenge, restricted mean survival time (RMST) has been strongly recommended for survival analysis in clinical literature due to its independence of the PH assumption as well as a more clinically meaningful interpretation. The RMST also aligns well with the estimand associated with the analysis from the recommendation in ICH E-9 (R1), and the test/estimation coherency. Currently, the Kaplan Meier (KM) curve is commonly applied to RMST related analyses. Due to some drawbacks of the KM approach such as the limitation in extrapolating to time points beyond the follow-up time, and the large variance at time points with small numbers of events, the RMST may be hindered.

**Methods:**

The dynamic RMST curve using a mixture model is proposed in this paper to fully enhance the RMST method for survival analysis in clinical trials. It is constructed that the RMST difference or ratio is computed over a range of values to the restriction time τ which traces out an evolving treatment effect profile over time.

**Results:**

This new dynamic RMST curve overcomes the drawbacks from the KM approach. The good performance of this proposal is illustrated through three real examples.

**Conclusions:**

The RMST provides a clinically meaningful and easily interpretable measure for survival clinical trials. The proposed dynamic RMST approach provides a useful tool for assessing treatment effect over different time frames for survival clinical trials. This dynamic RMST curve also allows ones for checking whether the follow-up time for a study is long enough to demonstrate a treatment difference. The prediction feature of the dynamic RMST analysis may be used for determining an appropriate time point for an interim analysis, and the data monitoring committee (DMC) can use this evaluation tool for study recommendation.

## Background

The log-rank test is one of the commonly used methods for survival analysis, and is considered the most powerful tool to compare two survival curves under the PH assumption. However, in the IO therapy trials, observed data often present a clear deviation/violation of the PH assumption due to delayed effects, cure rate, crossing hazards, or a mixture of these phenomena [1].

The hazard ratio (HR) has been widely used to evaluate the treatment effect under the PH assumption. However, when this assumption is deviated, the resulting HR estimate as the metric for the treatment effect is difficult to interpret [2]. In an interview, Professor David R. Cox, the originator of the COX proportional hazard model, stated, “Of course, another issue is the physical or substantive basis for the proportional hazards model. I think that’s one of its weaknesses …” [3]. When the PH assumption is violated, a single HR may not be a good estimand or measurement for the treatment difference because the HR can often be hard to understand or interpret without PH [4]. In this case, the HR is not simply an average of the true HR over time, but instead is the weighted average of the HR over time on the log scale [5]. In the Cox-regression model, the weights depend on the censoring distribution and different settings of accrual, follow-up, and early dropout in randomized clinical trials. Thus, this could lead to different trial results and parameter estimates even if the underline survival curves are identical no matter how large the sample sizes might be [5, 6]. In addition, median survival time may not be estimable due to long-term survival. When designing a clinical trial under non-PH, most likely we will mis-specify how the difference between groups varies over time due to a lack of PH, therefore, the HR estimation procedure may not be able to effectively detect a true difference between groups. Thus, the HR estimation procedure is a non-robust measure of the difference between two survival curves under non-proportional hazards [6]. Even when the PH assumption is a reasonable assumption, the HR may not be a useful summary of the treatment difference for decision making due to lack of the reference hazard value and the same HR value may have a completely different interpretation due to different reference hazard values [6]. Even though alternative methods have been suggested to replace the log-rank test for improving the power, without resolving the HR interpretation issue, the estimand associated with the analysis is ambiguous, which clearly deviates from the recommendation in ICH E-9 (R1) [7] due to a violation of the test/estimation coherency [8].

Chappell and Zhu [9] explored many different ways in describing differences in survival curves, including HR, median survival, ratio of landmark survival, and ratio of restricted mean survival time (RMST, the life expectance within a given (restricted) time period). The authors concluded that none of these endpoints is uniformly superior and all should bear consideration. The choice of the method to compare 2 treatment regimens depends on scientific and clinical necessity.

The RMST offers an intuitive, clinically meaningful interpretation without any pre-assumed model assumptions, such as the PH assumption [10–13]. However, we need to restrict the comparison to some specified interval since the censoring prevents reliable estimation of the unrestricted mean lifetime. From a statistical point of view, the RMST is the mean length of survival time within a specific time window, which can be interpreted as the area under the survival curve within the window, and in practice, it can be viewed as the life expectancy within the specific time window. The procedure for estimating the difference in RMST between two treatment groups is always valid without any model assumptions. The RMST is more stable in comparison with the estimation of the median survival time [3], has a valid and clearly defined estimand, and can produce consistent results between the hypothesis testing and estimation. The RMST captures the survival curve within the considered time window which is more informative when a survival plateau is present on the long term, for example in several immunotherapy RCTs; whereas the median used in HR estimate is unable to detect such a plateau in this right tail of the curve [14–16].

The RMST provides an absolute measurement based on a scale of time, whereas the HR reflects a relative parameter which does not have any unit. Trinquart, et al. [17] compared empirically the treatment effects measured by the HR and by the difference (and ratio) of RMST in 54 oncology randomized trials. In summary, on average, the HR provided significantly larger treatment effect estimates than the ratio of RMST (as calculated at the latest follow-up time). The authors recommend RMST-based measures be routinely reported in randomized trials with time-to-event outcomes.

Huang and Kuan [5] did extensive simulations under various scenarios and design parameter setups and compared the log-rank test and RMST-based test methods. When there is an evident separation favoring one treatment arm at most of the time points across the Kaplan-Meier survival curves, the log-rank test is generally a powerful test, but the RMST test has a similar performance. However, when the PH assumption is violated for scenarios where a late separation of survival curves is observed, the RMST-based test has better performance than the log-rank test when the pre-specified truncation time τ for defining the RMST is reasonably close to the tail of the observed curves.

RMST is robust with good interpretation for any survival distribution. Zhao, et al. [18] suggested using an RMST curve based on the RMST over time to quantify/evaluate the difference between two RMST curves within a specific time window. As an early noted restriction of RMST due to right censoring, the RMST inference is only available for the time period up to the minimum of the latest follow-up for the two groups. The statistical inference can be obtained for the RMST difference within a prespecified time window. However, the choice of the time window is crucial, and the resulting confidence bands for the difference of RMST curves depends on the choice of this time interval. Similarly, this issue also applies to the case for the simultaneous inference about the difference of two survival curves or hazard ratio.

The integration under the KM curve from the beginning of the study through a pre-specified time point is commonly used to calculate the RMST. However, the KM method shows some limitations in practical applications. First, the curve may not be able to extrapolate to time points beyond the follow-up time. The estimates may have large variance at time points towards the tail due to small numbers of patients at risk. As pointed out by Peto, et al. [19] the standard error can be underestimated in this long flat region. The curve is also estimated as a step function, which is biologically unrealistic. These drawbacks of the KM method limit the RMST performance because the survival comparison can only be estimated until the last event time or observation time, and a potential large variance for the estimates is presented at the last time point. In a typical clinical study, some prediction or extrapolation is needed and can be useful, for example, determining when the next interim analysis should be performed, or assessing if additional follow-up time in a study is needed to demonstrate a treatment difference. However, the KM method cannot fulfill this goal and so a parametric method is more desired.

To address these challenges from KM-based RMST method, in this paper we applied a flexible parametric mixture model to estimate the survival curves. Therefore, a dynamic RMST curve is constructed over any given time window of interest for the clinical study. A mixture model can fully take advantage of a parametric form for inference without limitation of the follow-up time. Liao and Liu [20] showed that the mixture models have good flexibility, i.e. the estimated survival curves can be very close to KM curves, to fit survival data from several oncology studies. The paper is organized as follows. In Section 2, we introduce the RMST method, and describe how to derive the dynamic RMST curves from the mixture Weibull models, where the RMST difference or ratio is computed over a range of values to the point of restriction τ, tracing out a curve over time. Three real datasets are used to illustrate the performance of the proposed dynamic RMST curves in section 3. Summary and discussions are provided in section 4 with a conclusion in section 5.

## Methods

Following Royston and Parmar [21], the RMST, μ(*τ*), of a random variable T is the mean of the survival time X = min (T, τ) limited to some horizon τ > 0. It equals the area under the survival curve S (t) from 0 to τ:

μ(*τ*) = E (X) = E [min(T, τ)]= $$ {\int}_0^{\tau }S(t) dt. $$

When T is years to death, we may think of μ(*τ*) as the ‘τ -year life expectancy’. The variance, var(X), of the restricted survival time X, is calculated as

$$ \mathit{\operatorname{var}}(X)={RSDST}^2=E\left({X}^2\right)-{\left[E(X)\right]}^2=2{\int}_0^{\tau } tS(t) dt-{\left[{\int}_0^{\tau }S(t) dt\right]}^2 $$

The restricted standard deviation (RSDST) is $\sqrt{\mathit{\operatorname{var}}(X)}$. In a two-arm clinical trial with survival functions *S*_0_ (t) and *S*_1_ (t) for the control and treatment arms, respectively, the difference in RMST between arms (treatment – control), Δ(τ), is given by

$$ \Delta  \left(\tau \right)={\int}_0^{\tau }{S}_1(t) dt-{\int}_0^{\tau }{S}_0(t) dt={\int}_0^{\tau}\left({S}_1(t)-{S}_0(t)\right) dt $$

i.e., Δ(휏) is the area between the survival curves. The delta method can be used to calculate the variance *var*(*Δ*(*τ*)) and then construct the confidence interval for the RMST difference Δ(휏) using the normal approximation. The treatment effect estimation that corresponds to the RMST based test can be performed by constructing a pointwise two-sided 1-α interval based on the standard normal approximation as

$$ \varDelta \left(\tau \right)\pm {z}_{1-\alpha /2}\sqrt{\operatorname{var}\left(\Delta \left(\tau \right)\right)} $$

where *z*_1 − *α* ∕ 2_ is the 100(1 − α/2)-th percentile of the standard normal distribution. Note that a simultaneous confidence interval for RMST differences may also be constructed using a similar procedure proposed by Zhao et al. [18].

The ratio of RMST between arms, *θ*(*τ*), is given by

$$ \theta \left(\tau \right)=\frac{\int_0^{\tau }{S}_1(t) dt}{\int_0^{\tau }{S}_0(t) dt} $$

Similarly, the confidence interval for *θ*(*τ*) can be constructed by calculating the confidence interval for log(*θ*(*τ*)) using delta method and then transforming back. In theory, a simple parametric model such as the Weibull, or Gompertz, or three parameter Gamma can be used to estimate RMST. However, a simple parametric model may not well fit the complex survival curve often seen in recent IO development. Several methods of estimating RMST are available [22] and discussed in literature, including direct integration of Kaplan-Meier survival curves, a jackknife method, and the Royston and Parmar’s modelling using a smoothing spline for the log-hazard function, and more recently the trapezoidal rule approach [23]. Note that KM uses a step hazard rate function. As pointed out in Royston and Parmar’s paper, the direct integration of Kaplan-Meier curves may be unreliable. The jackknife method has the advantage of being non-parametric but the drawback of being relatively slow to compute, which makes it cumbersome when simulation with many replicates is needed. Tian’s presentation [24] indicated that the performance of these RMST methods depends on the selection of τ. However, pre-specification of τ is not always easy. Using a large *τ* may not always be better because the separation of survival curves may not increase over time in long-term trials. In addition, a KM survival curve is not defined beyond the largest follow-up time. Therefore, in applications we can only pre-specify the τ as the minimum of the longest follow-up times for treatment groups, which will be data dependent.

To avoid these difficulties, we consider a flexible survival function as defined from the mixture of three components of Weibull [20]

$$ S(t)={p}_1\exp \left[-{\left(\frac{t}{\lambda_1}\right)}^{k_1}\right]+{p}_2\mathit{\exp}\left[-{\left(\frac{t}{\lambda_2}\right)}^{k_2}\right]+\left(1-{p}_1-{p}_2\right)\mathit{\exp}\left[-{\left(\frac{t}{\lambda_3}\right)}^{k_3}\right] $$

Liao and Liu [20] have demonstrated that the mixture model with 3 components of Weibull distribution fulfills the needs for modeling the delay effect or survival sudden drops, cure rate, or long term survival which are often observed in recent IO development. The advantage of using the mixture Weibull models includes: a) it is flexible and can produce a survive curve almost the same as the KM fitting; b) it is fully parametric which allows predicting future events, survival probability, and hazard function. In addition, the estimated hazards and survival curves are smooth functions as compared to the step functions from the Cox model or nonparametric estimators such as the KM method. In real applications, if a prior knowledge is available for the number of subgroups/components based on composition of the study population, then this knowledge should be used to determine the number of components for the mixture distribution. Huang, et al. [25] used this mixture model to estimate the progression free survival (PFS) and overall survival (OS) - functions based on an interim dataset and showed that the models can predict the final PFS and OS survival curves very well.

With the parametric components, the RMST across the entire time space, i.e., (0, ∞), can be estimated directly from the mixture Weibull estimates using ${\int}_0^{\infty }{e}^{-a{x}^b} dx=\frac{1}{b}{a}^{-\frac{1}{b}}\Gamma \left(\frac{1}{b}\right)$ and ${\int}_0^{\infty }{x}^n{e}^{-a{x}^b} dx=\frac{1}{b}{a}^{-\frac{n+1}{b}}\Gamma \left(\frac{n+1}{b}\right)$, where Γ(*p*) is a gamma function. In fact, the RMST *μ*(*τ*) can be estimated for any given *τ* using the incomplete gamma function $\gamma \left(s,x\right)={\int}_0^x{t}^{s-1}{e}^{-t} dt$, which can be computed using the r-function. The standard error and pointwise confidence intervals for *μ*(*τ*) can also be obtained from delta method.

In applications, the estimated *μ*(*τ*) and its pointwise confidence intervals provide dynamic views for the treatment effects over a different time window *τ*. As compared to the KM-based RMST analysis, this dynamic RMST analysis provides several advantages such as 1) a straightforward estimate calculation, 2) a better control on variability since the KM method may have large variance towards the tail, 3) easy implementation when adjusted for covariates, and 4) the availability of using RMST across the entire time space. Specifically, the last point of advantages would be useful for checking whether the follow-up time is long enough to demonstrate a treatment difference or to reach the maximum of the treatment effect (e.g., the estimated difference can be better later if follow up is longer) by checking the direction of the dynamic RMST difference or RMST ratio for a stabilized treatment effect; and it is useful for determining a time point for interim analysis by picking the time where the dynamic RMST difference or RMST ratio crossing a pre-defined acceptable treatment effect or for predicting future trends. In general, the timepoint selected for the analysis is very critical and is not a purely statistical issue. It should be chosen based on clinical consideration for the treatment and disease area such as how long we should follow the patients (for example, 3 years) to obtain enough information to assess the treatment benefit and harm. The tools mentioned here can be used to help understanding how feasible and reasonable of this choice. This piece of information will be explored in the three real datasets in the next section.

It should be noted that although the RMST curves based on the mixture models can be calculated over the entire time space in theory but we may not want to extend the estimated RMST too far away from the study follow-up to avoid too much extrapolation. Huang, et al. [25] extrapolated the PFS and OS survival curves using the mixture model based on the interim data and predicted the final PFS and OS survival curves very successfully. Based on the data maturity on which the mixture model was built, the amount of extrapolation can be varied. As pointed out in Liao and Liu [20], some practical guidelines are provided by Pocock et al. [26] and Gebski et al. [27] about curtailing the KM plot when the risk set is too small in applications. We apply the methods to a few real oncology trials in the next section to illustrate the flexibility and advantage of the mixture-model-based RMST analysis as compared to KM-based RMST.

## Results

### EORTC 1684 study

**Fig. 1:**
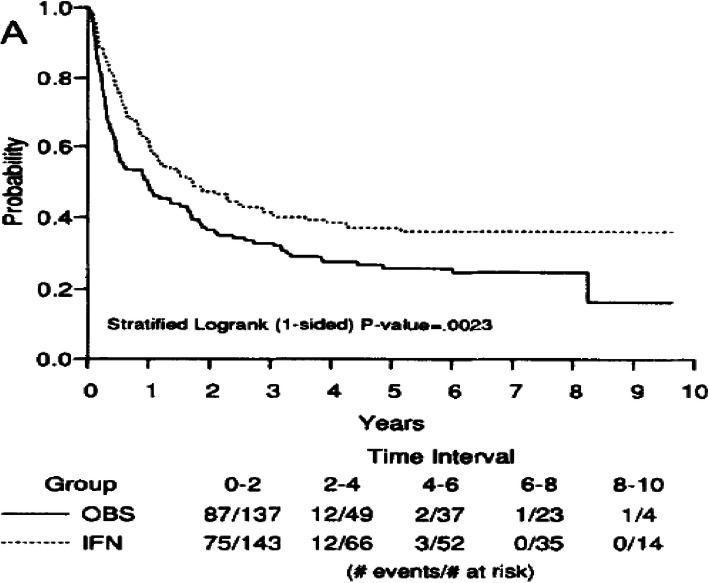
The screenshot of the KM survival curves for the EORTC 1684 study data [28]

Consider the EORTC 1684 [28] data (re-constructed) from a 48-week randomized controlled study of IFN alpha-2b versus control for patients who had histologically proven primary cutaneous melanoma without prior systemic adjuvant therapy and without evidence of distant metastatic disease. The primary analysis of overall survival by intent to treat (ITT) included data from 280 randomized patients (143 IFN patients and 137 observation patients). The KM survival curves for both treatment arms are screenshotted in Fig. 1 from the published paper for convenience, which shows an early treatment separation in a good direction and feasible log-term survival. The overall median survival time was 3.82 years (95% confidence interval [CI], 2.34 to 7.08) for IFN recipients as opposed to 2.78 years (95% CI, 1.83 to 4.03) for the control. Based on the Cox PH model analysis, the difference between treatment and control groups was highly significant with *p* = 0.0023 (one-sided), adjusting for disease status at randomization. In this example, the PH model is reasonable because a check of the PH assumption using the cox.zph() test of Grambsch and Therneau [29] had a *p*-value of 0.489 (*p*-value < 0.05 indicates violation of PH).

**Fig. 2:**
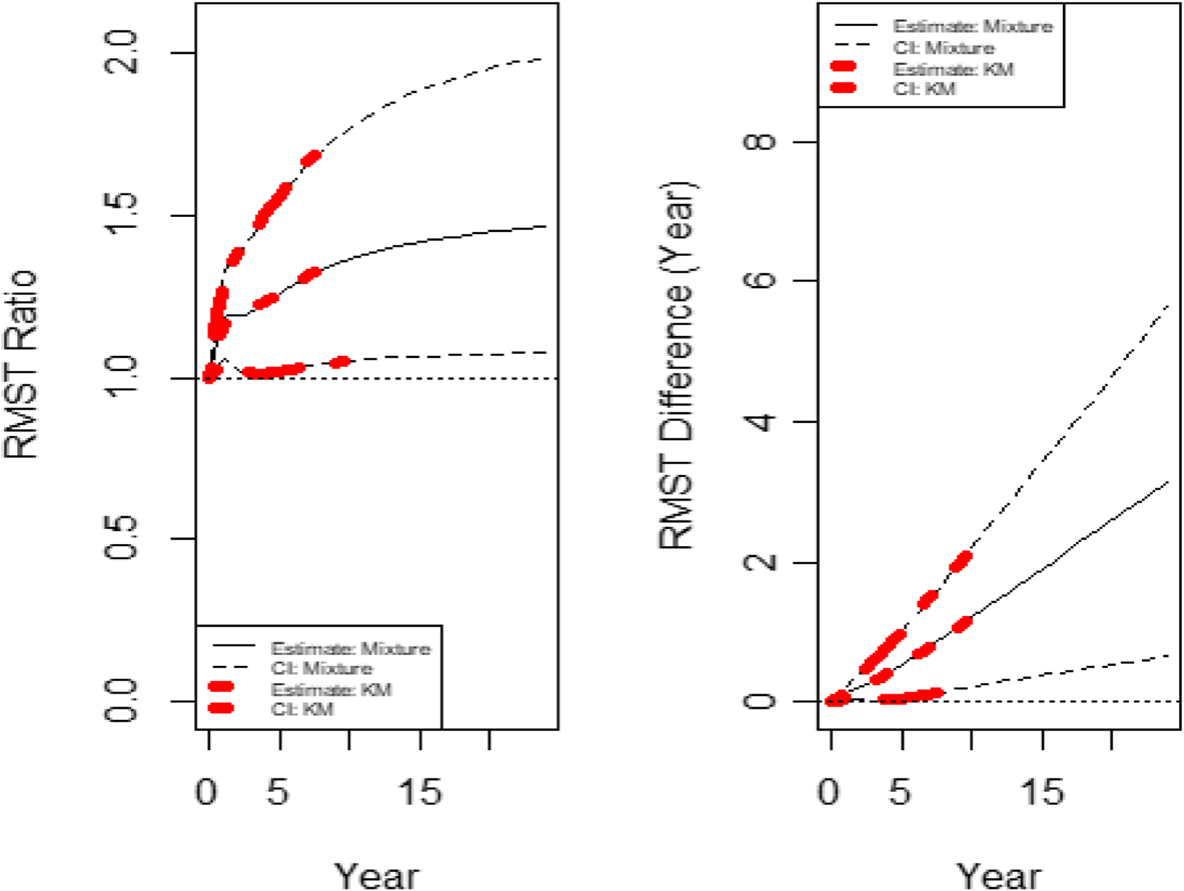
The dynamic RMST ratio and difference curve for the re-constructed EORTC 1684 study data

With the default *τ*= 9.496 years (the minimum of the largest observed time on each of the two groups), the estimated RMST value is 4.318 years and 3.177 years for the experimental arm and the control arm, respectively. To assess the treatment effect, the RMST curve with its 95% pointwise confidence interval based on KM curve using the R-package “survRM2” [30, 31] and the mixture model method in this paper for both RMST ratio and RMST difference are presented in Fig. 2. Note that RMST curve using KM method can only be reached to the min-max of the event time in the two treatment arms mentioned in previous section. However, the RMST curve using mixture model can be constructed in any time interval in principle, but we do not recommend having RMST curve to infinity. Both RMST ratio and difference analysis in Fig. 2 indicate that the treatment arm has benefit (based on the lower bound of CI above 1 or 0) from the beginning of the study with an early survival curve separation and maintains the benefit trend through all follow-up times. Note that the RMST curve using KM method and the mixture model aligned very well. Note that the *p*-value for RMST difference and ratio at the default *τ*= 9.496 years is 0.018 and 0.020, respectively.

Figure 2 shows the dynamic RMST ratio and difference curve, where RMST difference has an increasing trend all the time but the RMST ratio seems plateaued around 1.5. This implies that the treatment increases the life expectancy about 50% comparing to the control arm. Even though this is a positive study, if the follow-up time for this study was a little longer, the RMST ratio may have stabilized which could be valuable information for the drug label. Thus, in practice we may have both the RMST difference and RMST ratio curves generated for assessing the treatment effect. The RMST ratio may provide an alternative measure for the HR commonly used in clinical trials. Note that the RMST difference is in a clinical meaningful scale while the RMST ratio is unitless. All this dynamic information from RMST curve is missed from a log-rank based approach.

The RMST constructed here is based on the re-constructed data where the time to events or censoring are essentially represented by the graph of the survival curve without individual patient data. Therefore, the quality of this reconstruction could have an impact on the quality of the statistics carried out thereafter.

### CheckMate141 OS data

Consider the re-constructed data from a randomized, open-label, phase 3 trial of nivolumab (Opdivo®, Bristol-Myers Squibb) for patients with recurrent squamous-cell carcinoma of the head and neck [32]. A total of 361 patients were randomized in a 2:1 ratio: 240 patients received intravenous nivolumab, and 121 patients received a standard, single-agent therapy of the investigator’s choice (control), with stratification according to receipt of previous cetuximab therapy (yes or no). The primary endpoint was overall survival.

**Fig. 3:**
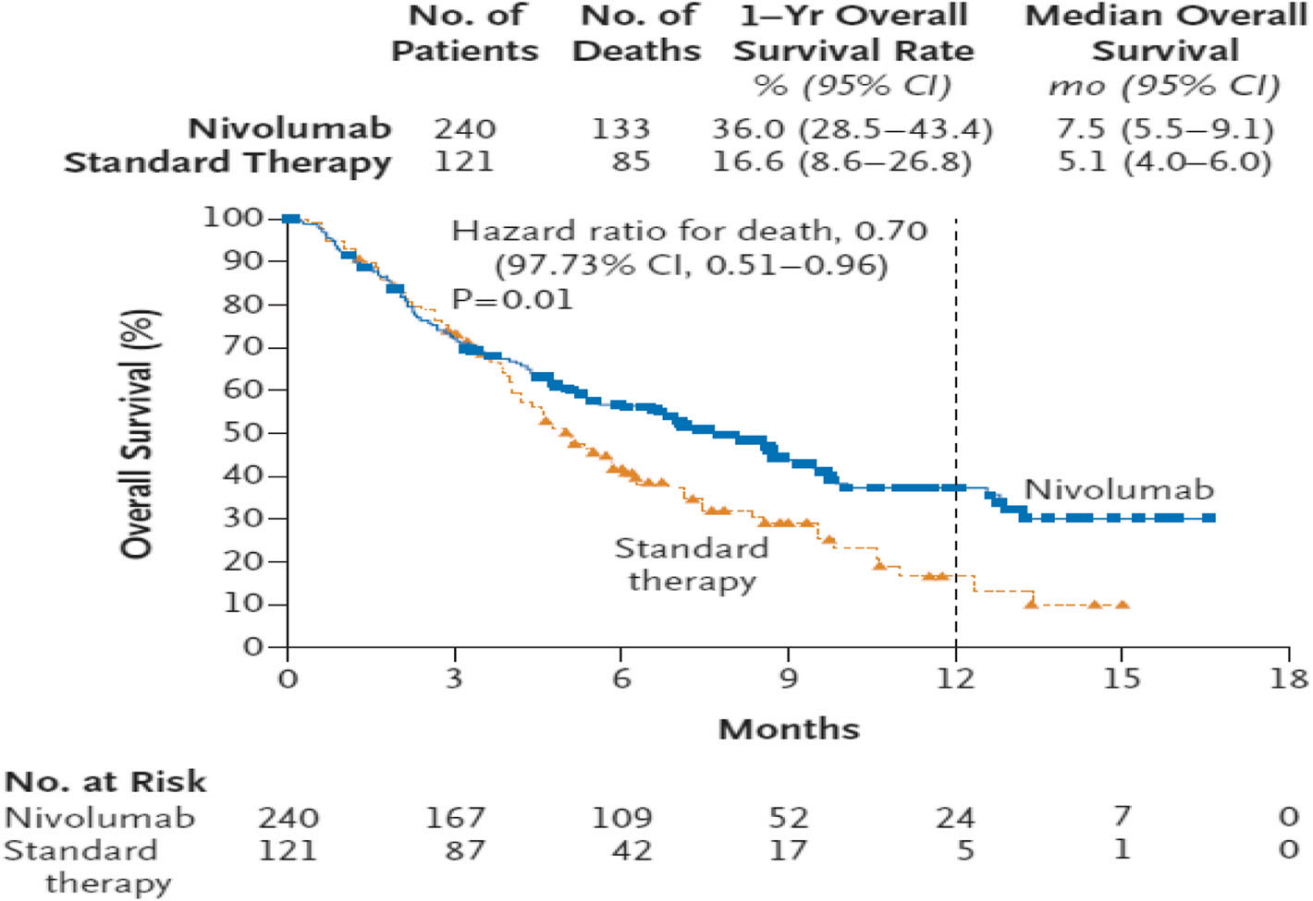
The screenshot of the KM survival curves for the CheckMate141 OS data [32]

The KM survival curves for both treatment arms are screenshotted in Fig. 3 from the published paper for convenience, which shows a delayed treatment effect and some signs of long-term survival. The median overall survival was 7.5 months (95% CI, 5.5 to 9.1) in the nivolumab group versus 5.1 months (95% CI, 4.0 to 6.0) in the control group. Overall survival was significantly longer with nivolumab than with control. The hazard ratio for death from Cox PH model was 0.70 with 95% CI, 0.51 to 0.96. The stratified log-rank test had a *p* value of *p* = 0.01. In this example, while the PH model assumption passed the *p*-value 0.05 threshold a check of the PH assumption using the cox.zph() test of Grambsch and Therneau [29] yielded a p-value of 0.061. Given the long delayed effect observed in the survival curve and marginally significant test of Grambsch and Therneau [29], the PH assumption may be in doubt.

**Fig. 4:**
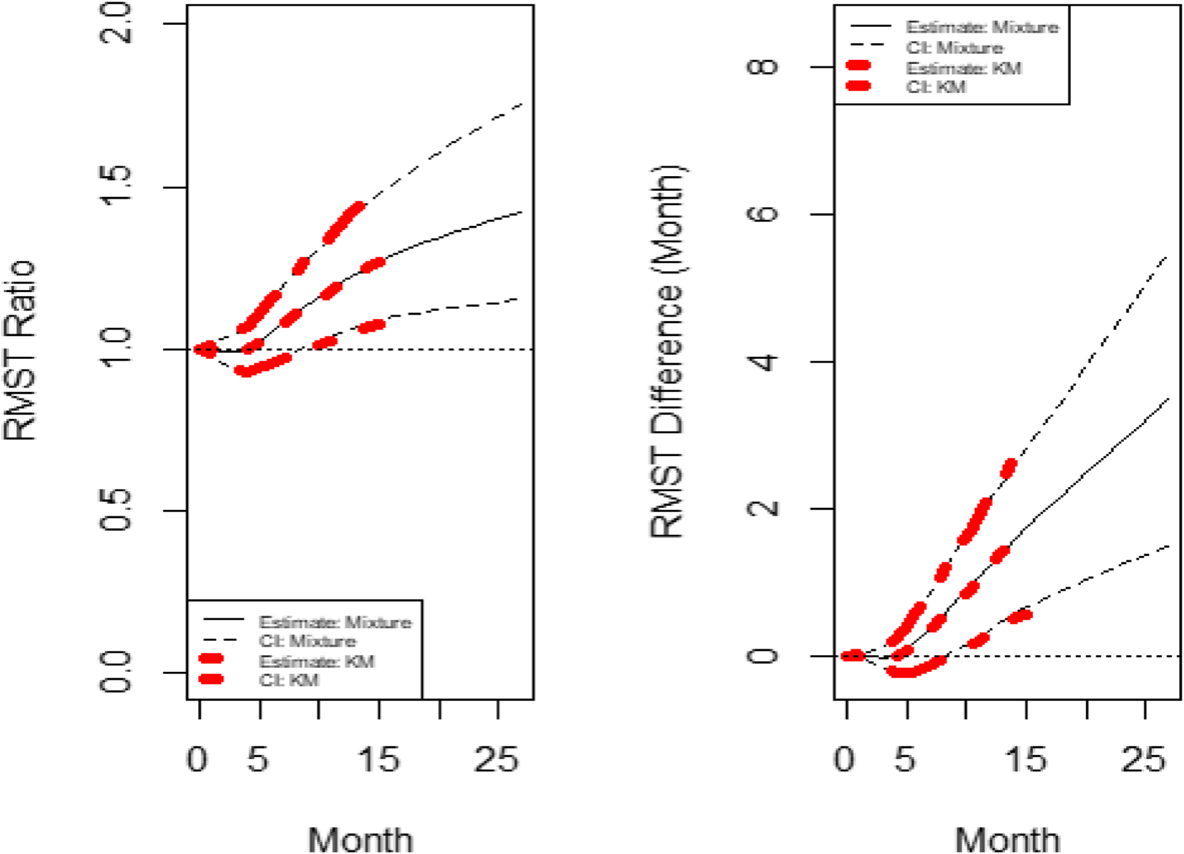
The dynamic RMST ratio and difference curve for re-constructed CheckMate141 OS data

With the default *τ*= 15 months (the minimum of the largest observed time on each of the two groups), the estimated RMST value is 8.189 months and 6.468 months for the experimental arm and the control arm, respectively. Similar to the first example, the RMST curve using both KM and the mixture model is shown in Fig. 4. Figure 4 indicates that there was no treatment benefit, as a matter of fact, it was identical to the control arm, up to about 4 or 5 months. Then it starts to demonstrate the treatment benefit after this time point. This dynamic feature of RMST curves clearly shows a delayed effect, which cannot be seen using the log-rank test approach. Note that again, the RMST curve for KM and mixture model approaches aligned very well. Note that the *p*-value for RMST difference and ratio at the default *τ*= 15 months is 0.004 and 0.005, respectively.

Figure 4 shows the dynamic RMST ratio and difference curve, where the RMST ratio curve does not plateau at any time due to the apparent cure in treatment group. Thus, the follow-up time is adequate for this study.

### CheckMate 057

**Fig. 5:**
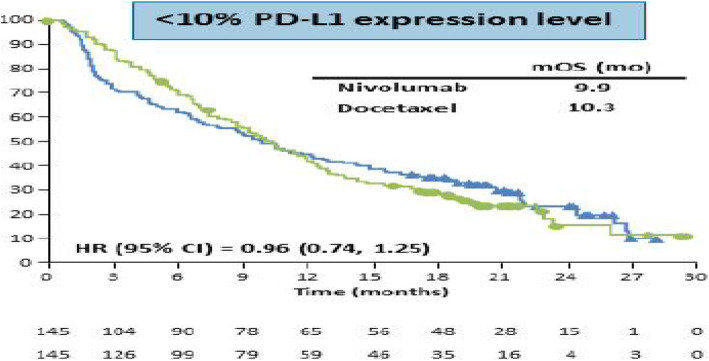
The screenshot of the KM survival curves for the CheckMate 057 data [33]

Consider the re-constructed data from the randomized, open-label, international phase 3 study for Nivolumab for patients with non-squamous non–small-cell lung cancer (NSCLC) that had progressed during or after platinum-based doublet chemotherapy [33]. From November 2012 through December 2013, 292 patients were randomly assigned to receive nivolumab, and 290 were randomly assigned to receive docetaxel (SOC control group). The primary endpoint was overall survival. Tumor PD-L1 protein expression was assessed retrospectively in prospectively collected specimens. There were 145 patients in each treatment group for PD-L1 expression level less than 10%. For illustration purpose, the analysis was conducted on this PD-L1 expression level less than 10% subgroup. The median OS was 9.9 and 10.3 months for SOC group and Nivo group, respectively, and the HR 0.96 (95% CI: 0.74, 1.25) with a *p*-value of 0.6199. The KM survival curves for both treatment arms are screenshotted in Fig. 5 from the published paper for convenience, which shows a crossing and diminishing treatment effect. In this example, the PH model may not be appropriate because a check of the PH assumption using the cox.zph() test of Grambsch and Therneau [29] had a p-value of 0.016.

**Fig. 6:**
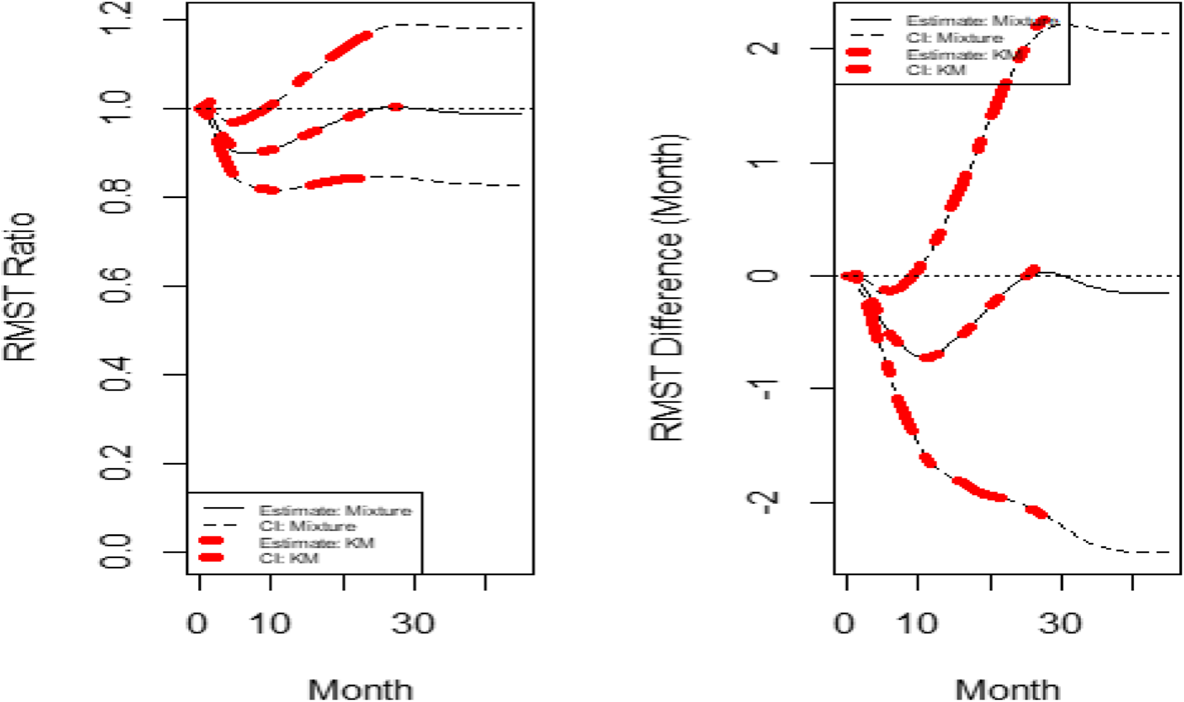
The dynamic RMST ratio and difference curve for the re-constructed CheckMate 057 data

With the default *τ*= 27.752 months (the minimum of the largest observed time on each of the two groups), the estimated RMST value is 12.667 months and 16.605 months for the experimental arm and the control arm, respectively. Figure 6 shows the dynamic RMST ratio and difference curve, where the RMST curve for prior to about 10 months shows the control arm has a better performance. Then, the treatment arm improves the benefit and in the long run, there is no clear difference between the two treatments. Note again, the RMST curve for KM and mixture model approaches aligned very well. Note that the *p*-value for RMST difference and ratio at the default *τ*= 27.752 months is 0.955 and 0.955, respectively.

Figure 6 clearly indicates a crossing effect at approximately 10 months with control arm being better. RMST ratio and difference indicates the plateau since all patients died. It also indicates the follow-up time can be a few months shorter but reach the same conclusion. Again, this very useful information cannot be obtained from the log-rank HR approach.

## Discussions

RMST provides a clinically meaningful and easily interpretable measure for survival clinical trials. Unlike the log-rank HR summary which heavily relies on the PH assumption, the RMST is always valid regardless of the PH assumption. The RMST always aligns well with the estimand associated with the analysis from the recommendation in ICH E-9 (R1), and the test/estimation coherency. The method has been recommended in recent publications [3, 5, 17, 18]. Traditionally, the RMST is estimated from KM curves, therefore it is limited to the time window up to the study follow-up. In this paper, a mixture Weibull model is considered to estimate the survival curve which allows for construction of dynamic RMST at any given time point. This dynamic approach provides a useful tool for assessing treatment effect over different time frames for survival clinical trials.

Compared to the KM-based RMST analysis, this dynamic RMST approach provides advantages on computation for estimation and variance control. With parametric components, the estimated RMST curves can be extended to any time points. In applications, both RMST ratio and RMST difference were evaluated. As illustrated in the examples, when the RMST ratio reaches a plateau, it can be useful for checking whether the follow-up time for a study is long enough to demonstrate treatment difference. The prediction feature of the dynamic RMST analysis may also be useful for determining an appropriate time point for interim analysis, and an evaluation tool for study recommendation from DMC.

With the mixture model approach, the parametric form allows easy adjustment of covariates. It is common that the treatment effect can be impacted by baseline factors [34]. The ability to perform covariate-adjusted comparisons of two groups with respect to survival is important [35]. Due to the nature of the parametric format of the mixture model, it is relatively straightforward to accomplish covariate-adjusted comparisons of two groups for the RMST curve. With the mixture model approach, one may need to pay additional attention to select appropriate starting parameter values for the nonlinear fitting. Some suggestions for selecting the starting values can be found in Liao and Liu [20] and Liu and Liao [36].

When the event rate is low, the RMST ratio may result in extreme value close to 1 and therefore is not very intuitive. Instead, the ratio of restricted mean times lost, RMTL(τ)= $\tau -{\int}_0^{\tau }S(t) dt$ can be used [37]. Similar procedures can be used for RMTL(τ). In this paper, the confidence interval instead of *p*-value was used to make inference. However, the hypothesis testing approach can be used following the testing format in Royston and Parmar [21], or simultaneous testing with multiple time points [8], or the simultaneous CI approach with a fixed longest time point.

## Conclusions

The RMST provides a clinically meaningful and easily interpretable measure for survival clinical trials and it is also robust to be used for a survival distribution without any model assumptions such as the proportional hazards. As demonstrated in the examples, the proposed dynamic RMST approach provides rich information and is a useful tool for assessing treatment effect over different time frames for survival clinical trials. This dynamic RMST curve provides an innovated way for checking whether the follow-up time for a study is long enough to demonstrate a treatment difference. The prediction feature of the dynamic RMST analysis can also be applied to determine an appropriate time point for an interim analysis which is a useful evaluation tool for study recommendation from DMC.

## Data Availability

All data were re-generated using digital tools (https://automeris.io/WebPlotDigitizer/, Web Plot Digitizer) from the graphs in the published papers

## Abbreviations

ICH E-9 (R1): International Conference on Harmonization: addendum on estimands and sensitivity analysis in clinical trials to the guideline on statistical principles for clinical trials
DMC: data monitoring committee
IO: immuno-oncology
RMST: restricted mean survival time
KM: Kaplan Meier
PH: proportional hazard
PFS: progression free survival
OS: overall survival
HR: hazard ratio
CI: confidence interval
RMTL: restricted mean times lost
